# Oviposition Site Selection by the Dengue Vector *Aedes aegypti* and Its Implications for Dengue Control

**DOI:** 10.1371/journal.pntd.0001015

**Published:** 2011-04-12

**Authors:** Jacklyn Wong, Steven T. Stoddard, Helvio Astete, Amy C. Morrison, Thomas W. Scott

**Affiliations:** 1 Department of Entomology, University of California Davis, Davis, California, United States of America; 2 Naval Medical Research Center Unit-6, Lima, Peru; Colorado State University, United States of America

## Abstract

**Background:**

Because no dengue vaccine or antiviral therapy is commercially available, controlling the primary mosquito vector, *Aedes aegypti*, is currently the only means to prevent dengue outbreaks. Traditional models of *Ae. aegypti* assume that population dynamics are regulated by density-dependent larval competition for food and little affected by oviposition behavior. Due to direct impacts on offspring survival and development, however, mosquito choice in oviposition site can have important consequences for population regulation that should be taken into account when designing vector control programs.

**Methodology/Principal Findings:**

We examined oviposition patterns by *Ae. aegypti* among 591 naturally occurring containers and a set of experimental containers in Iquitos, Peru. Using larval starvation bioassays as an indirect measure of container food content, we assessed whether females select containers with the most food for their offspring. Our data indicate that choice of egg-laying site is influenced by conspecific larvae and pupae, container fill method, container size, lid, and sun exposure. Although larval food positively influenced oviposition, our results did not support the hypothesis that females act primarily to maximize food for larvae. Females were most strongly attracted to sites containing immature conspecifics, even when potential competitors for their progeny were present in abundance.

**Conclusion/Significance:**

Due to strong conspecific attraction, egg-laying behavior may contribute more to regulating *Ae. aegypti* populations than previously thought. If highly infested containers are targeted for removal or larvicide application, females that would have preferentially oviposited in those sites may instead distribute their eggs among other suitable, previously unoccupied containers. Strategies that kill mosquitoes late in their development (i.e., insect growth regulators that kill pupae rather than larvae) will enhance vector control by creating “egg sinks,” treated sites that exploit conspecific attraction of ovipositing females, but reduce emergence of adult mosquitoes via density-dependent larval competition and late acting insecticide.

## Author Summary

Controlling the mosquito *Aedes aegypti* is of public health importance because, at present, it is the only means to stop dengue virus transmission. Implementing successful mosquito control programs requires understanding what factors regulate population abundance, as well as anticipating how mosquitoes may adapt to control measures. In some species of mosquitoes, females choose egg-laying sites to improve the survival and growth of their offspring, a behavior that ultimately influences population distribution and abundance. In the current study, we tested whether *Ae. aegypti* actively choose the containers in which they lay their eggs and determined what cues are most relevant to that process. We also explored whether females select containers that provide the most food for their larval progeny. Surprisingly, egg-laying females were most attracted to sites containing other immature *Ae. aegypti*, rather than to sites containing the most food. We propose that this behavior may contribute to density-dependent competition for food among larvae and play a larger role than previously thought in regulating *Ae. aegypti* populations. We recommend that accounting for, and even taking advantage of, this natural behavior will lead to more effective strategies for dengue prevention.

## Introduction

Dengue viruses are transmitted to humans primarily by the mosquito *Aedes aegypti* and represent an increasing public health concern in tropical and subtropical regions worldwide. Because no vaccine or antiviral therapy is commercially available, controlling the mosquito vector is the only current means to prevent dengue outbreaks [1]. Contemporary control campaigns, rather than attempting to eradicate *Ae. aegypti*, aim to suppress mosquito populations below a threshold density at which they no longer support viral amplification [2]. Controlling adult mosquitoes is made challenging by the behavior of domestic *Ae. aegypti*. Adult *Ae. aegypti* rest inside homes, typically on clothing, curtains, bedspreads, and furniture, items that cannot be sprayed with residual insecticides [3]. Aerosol space sprays consist of small airborne droplets of insecticide designed to kill adult mosquitoes on contact, but difficulty in reaching indoor adult resting sites can limit their efficacy [4]. Even when space sprays are effective in reducing adult populations, effects are transient due to the continuing emergence of new adults or immigration from untreated areas [3], [5]. Insecticide-treated materials (curtains, water container covers, and bednets) have shown promise in reducing *Ae. aegypti* populations [6], [7], but the impact of these reductions on dengue transmission has not been determined.

Currently, the World Health Organization recommends directing routine *Ae. aegypti* control toward the immature stages [2]. *Ae. aegypti* females lay eggs singly just above the water line, often in man-made containers located in the home or yard (buckets, drums, tires, and vases, etc.) [8]–[10]. Eggs hatch when inundated, and larvae develop by filter feeding and browsing for microorganisms and organic detritus [11], [12]. Control approaches such as container removal (source reduction) and larvicide application aim to reduce the number of new emerging adults in the population [2]. Traditional models of *Ae. aegypti* assume that population dynamics are regulated predominantly by density-dependent competition for food during early larval stages and little affected by oviposition rates [13], [14]. Based on these models, some researchers have assumed that all containers suitable for larval development receive an excess of eggs, thereby leading all larvae to experience density-dependent competition [15]. This is the rationale behind targeted source reduction (a WHO-recommended control strategy) and the expectation that eliminating containers that, for example, produce 75% of adults will lead to a proportionate decrease in the overall adult population [15]–[17]. Much remains unclear, however, about the factors regulating *Ae. aegypti* adult production and how reducing, but not eliminating, containers will ultimately affect adult abundance.

In some mosquito species, female choice in oviposition site is adaptive and can influence population distribution and dynamics [18], [19]. Females can enhance survival and development of their offspring by selecting egg-laying sites that reduce exposure to predators and competitors [19], [20], or increase access to food [21], [22]. In general, understanding insect egg-laying decisions may provide additional insight into the factors affecting population regulation and aid in predicting how populations will respond to control measures [23]. Oviposition preferences by *Ae. aegypti* have been studied in the laboratory [24]–[31], but to a lesser extent in the field [32]–[36]. Research has typically involved varying one or two oviposition site factors at a time and observing the number of eggs laid in response (reviewed in [24]). Such studies reveal the types of abiotic and biotic stimuli potentially affecting oviposition, but yield limited information on the relative importance of these stimuli in nature [24], [37].

The goals of our study were to test whether free-ranging *Ae. aegypti* females make active choices regarding where they oviposit and to identify factors influencing oviposition. Although selective oviposition has been demonstrated using small oviposition traps in the field [32]–[35] or water-storage containers in an enclosure [36], we examined for the first time females' oviposition choices among naturally-occurring containers in homes throughout a large, dengue-endemic city. We also investigated the consequences of oviposition site selection for offspring fitness by testing whether females choose sites to maximize the amount of food available for their progeny. Food availability is known to affect components of mosquito fitness such as offspring survival, development time, and adult size [38]. Lastly, we considered the implications of selective oviposition behavior for *Ae. aegypti* population regulation and the success of targeted larval control strategies.

## Materials and Methods

### Study location

Our study was conducted in Iquitos (73.2°W, 3.7°S, 120 m above sea level), a city of approximately 380,000 people located in the Amazon Basin, Department of Loreto, Northeastern Peru [10], [39]–[41]. Rain falls during all months of the year and average temperature and relative humidity are fairly consistent [42]. During our study period from July 2007 to August 2009, mean monthly temperature ranged from 24.8°C (±1.1 SD) in June 2008 to 26.5°C (±1.1 SD) in December 2008. Average relative humidity ranged from 80.2% (±4.1 SD) in August 2007 to 86.2% (±4.4 SD) in April 2009. More detailed climate data for the years 2007 to 2009 are given in the Supporting Information (Table S1).

In response to the unreliable municipal water supply, Iquitos residents store water in containers [40]. Household containers are filled in three primary ways: 1) from spigots in the home or neighborhood (manually filled), 2) intentionally placed outside to collect rain water (rain-filled), and 3) filled with rain water as a result of being untended outside (unmanaged). Method of filling is correlated with the frequency of water turnover and amount of organic detritus present in containers, with manually filled containers kept the cleanest and unmanaged containers collecting the most organic material. Containers in Iquitos generally lack predators of larval *Ae. aegypti*, such as copepods or fish (ACM and JW, unpublished data), but do occasionally contain immature *Culex* which may act as competitors [10]. *Ae. aegypti* are reproductively active all year in Iquitos. Of the roughly 290,000 containers examined by Morrison et al. [10], 7.3% contained *Ae. aegypti* larvae and/or pupae.

### Observational study

#### Consent process

The households included in this study were identified through three ongoing, longitudinal cohort studies on dengue transmission dynamics approved by the University of California, Davis (Protocol #2006.14381, 2006.14405, 2007.15244) and Naval Medical Research Center Detachment (Protocol #NMRCD 2007.001, NMRC 2005.0009, NMRCD2007.007) Institutional Review Boards (IRBs). As described in detail by Morrison et al. [43], *Ae. aegypti* abundance surveys were conducted in private homes by two-person teams that administered a brief questionnaire to residents, counted the number of water-holding containers present on the property, inspected containers for immature *Ae. aegypti*, and collected adult mosquitoes using backpack aspirators. Entomological surveys required a verbal informed consent process in which the survey procedures were explained to residents and if they consented, the survey team was allowed into the household. Both IRBs approved verbal consent without written documentation because the survey form would indicate consent of the residents. Our oviposition study was approved by the local ministry of health (Dirección Regional de Salud -Loreto). The Naval Medical Research Center IRB determined that our study (Project #: PJT-NMRCD.032) did not meet the definition of human subject research.

#### Survey procedures

We conducted a large-scale survey to examine female oviposition choices among naturally-occurring containers in Iquitos homes. For nine weeks during July to September 2007 (collection period 1), seven weeks during May to July 2008 (collection period 3), and six weeks during October to December 2008 (collection period 4), we closely observed the number of *Ae. aegypti* eggs laid in containers within a subset of surveyed houses. Collection period 2 is described later. Each week, 3 to 6 houses having at least one *Ae. aegypti*-positive container were selected to be included in this study. For each of those houses, we visited 2 to 3 additional houses on the same block (matched in time and space) that had containers but no larvae, such that 9 to 18 total houses were visited per week. All surveyed houses, along with their associated entomological data, were geocoded using a geographic information system previously developed for Iquitos [39].

In each selected house, 2 to 4 inspectors examined the entire property (indoors and outdoors) for water-filled containers and used strips of brown paper towel to line the inside of containers (limited by homeowner permission) at the water line to collect eggs. The following characteristics were recorded on the first day: container size (circumference, capacity, and water volume), location and sun exposure, lid presence, fill method, insecticide treatment, conspecific larvae (abundance and estimated mean density), presence of conspecific pupae, and presence of immature *Culex* (Table 1). Insecticide treatment (temephos or pyriproxyfen) was scored depending on whether an insecticide sachet was present in containers; we did not determine how long sachets had been in containers or whether insecticidal activity was still active. The abundance of larval *Ae. aegypti* and the presence of larval *Culex* were noted by visual inspection without removing larvae. Larval *Ae. aegypti* estimates per container were categorized as: none, 1 to10, 11 to 50, 51 to 100, or >100 larvae. Estimated mean density of *Ae. aegypti* larvae was calculated by dividing the midpoint of the larval abundance category (or 200 in the case of >100 larvae) by water volume. Any pupae occurring in containers were collected daily and brought to the field laboratory to be counted and the emerging adults identified as either *Ae. aegypti* or *Culex spp*. If third instar *Ae. aegypti* larvae (determined by size and morphology) were present on the first day, up to 25 third instars were removed per container for starvation bioassays (described below) to assess food availability in containers [38], [44]. Otherwise, mosquito larvae were left undisturbed.

**Table 1:** Container characteristics recorded during oviposition survey in Iquitos, Peru, and regression parameters for oviposition models.

Variable	Levels	No. containers	Median (range)
Circumference	Continuous		126 cm (10; 540)
(Circumference)^2^	Continuous		15,791 cm^2^ (100; 291,600)
Location and sun exposure	Indoor (enclosed by roof and at least 3 walls)	134	
	Outdoor shade (exposed to sunlight <20% of day)	242	
	Outdoor sun (exposed to sunlight ≥20% of day)	215	
Lid	Absent	546	
	Present	45	
Fill method	Manually filled (from spigots, wells, etc.)	215	
	Rain-filled (intentional)	174	
	Unmanaged (unintentional rain water collection)	202	
Insecticide treatment	None	513	
	Temephos	46	
	Pyriproxyfen	32	
*Ae. aegypti* larvae	None	335	
	1–10 larvae, retained during survey	47	
	1–10 larvae, removed on day 1	22	
	11–50 larvae, retained during survey	33	
	11–50 larvae, removed on day 1	37	
	51–100 larvae, retained during survey	38	
	51–100 larvae, removed on day 1	17	
	>100 larvae, retained during survey	45	
	>100 larvae, removed on day 1	17	
*Ae.aegypti* pupae	Absent	454	
	Present	137	
Immature *Culex*	Absent	560	
	Present	31	
Collection period	1 - July to September 2007 (9 weeks)	222	
	2 - January to May 2008 (14 weeks)	202	
	3 - May to July 2008 (7 weeks)	93	
	4 - October to December 2008 (6 weeks)	74	

Paper strips were checked daily for three consecutive days to collect a representative sample of eggs laid within each house. Collections were conducted between 09:00 to 12:00 h to minimize disturbance of ovipositing females [45]. If eggs were present, new paper lining was exchanged. Papers with eggs were brought to the field laboratory to count eggs under a dissecting microscope at 20× magnification. Subsamples of collected eggs were hatched once a week to confirm their identity as *Ae. aegypti*. To prevent production of adult mosquitoes in sampled houses, containers with larvae were overturned or treated with pyriproxyfen at the conclusion of the 3-day survey.

During 14 weeks from January to May 2008 (collection period 2), we surveyed containers following the above procedures, with the exception that all larvae and pupae were removed using a net and/or turkey baster on the first day. Therefore, no immature mosquitoes were present in containers when females oviposited on the following three days, but the water was “conditioned” by the previous presence of immatures. All larvae were taken to the field laboratory, where they were enumerated to genus and instar. Up to 25 third instar *Ae. aegypti* per container were used for starvation bioassays as described below.

#### Data analysis

Regression analyses were conducted using R version 2.8.1 [46]. To check for spatial autocorrelation among containers surveyed in the same week as a potential confounder, we estimated Moran's I for egg counts using a Euclidean distance matrix with the APE package within R [47]. Because no spatial structure was evident, subsequent analyses did not take spatial coordinates into account. We attempted to include the density of adult female *Ae. aegypti* as a predictor variable in our models, but collections were too sparse (mean = 0.14±0.52 SD females per house) for meaningful analyses. Instead, using a separate chi square test, we examined whether the presence of *Ae. aegypti* larvae was independent from capture of adult females during the abundance survey.

To identify variables that best predicted whether or not female *Ae. aegypti* laid eggs in a container, a logistic regression model was fitted to our data (1 = container received eggs at least once during three days of observation, 0 = container received no eggs). Categories of *Ae. aegypti* larval abundance were further divided depending on whether larvae were retained during the survey or removed from containers on the first day. Collection period was included to control for time. The three measures of container size were collinear (circumference-capacity, Spearman's ρ = 0.86; circumference-water volume, Spearman's ρ = 0.65, capacity-water volume, Spearman's ρ = 0.85). Because the amount of space available for oviposition is determined by container circumference, we included circumference rather than capacity or water volume in our model. Larval abundance and estimated mean larval density also were collinear (Spearman's ρ = 0.92). Larval abundance was used because it provided a better model fit to the data. Starting with a saturated model including all variables listed in Table 1, we employed a log-likelihood test to eliminate, stepwise, the non-significant variable with the greatest χ^2^ p-value (2× log-likelihood of current model–2× log-likelihood of previous model ∼χ^2^, df = 1, p>0.10). If the final model included a variable with more than two levels, Tukey's multiple comparisons were applied using the MULTCOMP package [48] to identify differences in level effects.

Only containers receiving eggs were included in the analysis to identify variables influencing the number of eggs laid in containers. Negative binomial regression was performed using the MASS package [49]. Our response variable was the mean number of eggs laid per container per day, rounded to the nearest integer. To more closely examine the association between egg abundance and container size, we included both container circumference and (circumference)^2^ as predictor variables in the model. As with the logistic regression model, containers were classified according to larval abundance and whether or not larvae were removed on the first day, and to collection period to control for time. Model selection was based on the log-likelihood test. To confirm that model assumptions were met, deviance residuals were plotted against: (1) fitted values, (2) each explanatory variable included in the model, (3) each explanatory variable eliminated from the model, (4) survey date, and (5) spatial coordinates [50].

### Starvation bioassays

We measured larval resistance to starvation (RS, number of days larvae survive without food) as an indirect measure of per capita food availability in containers [38]. In general, mosquito larvae that consume more food are able to store more energy reserves and resist starvation longer [13], [44]. During the above-described survey of Iquitos containers, 5 to 25 third instar larvae were removed from containers in the field and transferred to individual plastic cups (5 cm diameter×6 cm height) filled to 2/3 capacity with bottled drinking water. Third instars were used for bioassays because fourth instar *Ae. aegypti* frequently pupate when starved [44]. Cups were placed indoors in our field laboratory, where larvae were exposed to natural light and temperature. Water was changed every two days to prevent accumulation of waste and microbial growth [51]. Time to death (in days) was recorded for each larva. Because starvation times were not distributed symmetrically for larvae from each container, the median larval RS was used as the measure of central tendency for the data for each container.

Spearman rank correlation was used to identify any association between larval RS and egg density (mean eggs laid per day/circumference). Data were stratified according to whether or not all larvae had been removed from containers on the first survey day. To account for potential effects of larval abundance and container size, data also were stratified by larval abundance (≤50 larvae vs. >50 larvae) and container capacity (≤20 L vs. >20 L).

### Experimental study

For 12 weeks during June to August 2009, we carried out an experimental study manipulating both the presence of conspecific larvae and accumulation of organic material in containers and recorded oviposition by wild females. This experiment was replicated in three central Iquitos residences, the courtyard of our field laboratory and in the yards of two other houses selected based on the consistent presence of *Ae. aegypti* and homeowner willingness to participate. At each residence, three identical 6-liter blue plastic buckets (20 cm diameter×23 cm height) were placed close to one another (0.5 m apart) to minimize differences in container position. Hourly at each house, ambient temperature and relative humidity were recorded using a Hobo® ProV2 data logger (U23-001; Onset Computer Corporation, Pocasset, MA) and water temperature was recorded in one container per house using a Hobo® Pendant logger (UA-002-64).

We created three container treatments: A (unmanaged, with larvae), B (unmanaged, no larvae), and C (manually filled, no larvae). Unmanaged containers (A and B) were filled with four liters of tap water and allowed to accumulate organic debris for 12 weeks, whereas manually filled containers (C) were cleaned and refilled with new tap water every other day. Fifty first instar *Ae. aegypti* larvae were introduced into treatment A containers every two weeks starting on the first day. Oviposition was monitored by lining the inside of buckets with strips of brown paper towel to collect eggs. Every second day, papers were exchanged and the number of eggs counted as described above. On egg collection days, we also temporarily removed larvae from treatment A containers to determine their developmental stage and count them. Larvae were then returned to the container from which they originated.

To estimate the accumulation of organic detritus and bacterial growth in unmanaged containers, a thoroughly mixed water sample was measured for cloudiness using a turbidity tube [52] and dissolved oxygen content using an Ecological Test Kit (Rickly Hydrological Company, Columbus, OH). Water samples were returned to containers after testing. In all containers, tap water was added every few days to replace water lost to evaporation. Any pupae were removed to prevent emergence of adult *Ae. aegypti*.

#### Data analysis

Due to repetitive sampling, effects of treatment (A, B, or C), house, and week on the number of eggs laid per week (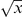 transformed) were analyzed by repeated measures analysis of variance (RM ANOVA). RM ANOVA was also used to examine effects of treatment, week, and house on water turbidity (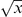 transformed) and dissolved oxygen content (χ^3^ transformed). RM ANOVAs were carried out using PROC MIXED in SAS version 9.2 [53] and transformations were performed to meet ANOVA assumptions.

## Results

### Observational study

**Figure 1:**
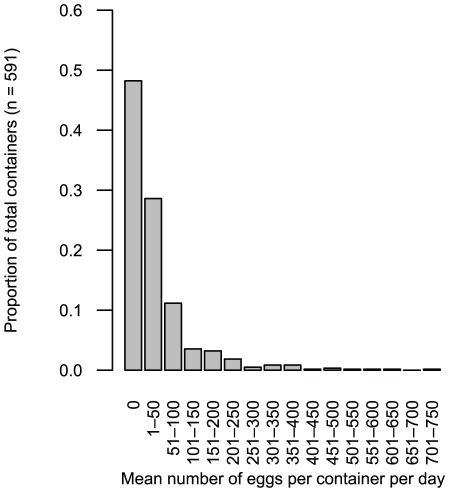
Frequency distribution of *Ae. aegypti* eggs. Number of eggs collected per day in naturally-occurring containers throughout Iquitos, Peru (n = 591 containers).

We monitored oviposition in 591 containers in 448 households across Iquitos. *Ae. aegypti* eggs were deposited in 51.8% of surveyed containers (306 of 591). Egg counts per container per day were strongly skewed, with the majority of containers receiving 0 to 50 eggs (median = 2, mean = 41), and a few containers receiving hundreds of eggs (Figure 1). All mosquitoes reared from collected eggs were *Ae. aegypti*, which we found to be the only *Aedes* species present in domestic containers throughout Iquitos. The presence of *Ae. aegypti* larvae in households was independent from whether or not adult females were caught during entomological surveys (χ^2^ = 1.897, df = 1, p = 0.169). *Culex* mosquitoes were occasionally present in the same containers (5.2% of all containers surveyed, 11.3% of *Ae. aegypti*-positive containers), but were easily distinguished by morphology. We did not find any containers colonized only by *Culex*.

After controlling for collection period, three variables were significant predictors of whether females laid eggs in containers: *Ae. aegypti* larvae, exposure to sunlight (≥20% of day), and absence of a container lid (Table 2). The probability of oviposition increased when sites held conspecific larvae (β = 1.658; 95% CI = [1.286, 2.030]; p<0.001), an effect which remained consistent regardless of larval abundance or whether larvae had been removed from containers during the day(s) prior to egg collection. Containers located outside and exposed to sunlight (≥20% of the day) were more likely to receive eggs compared to indoor containers (β = 0.601; 95% CI = [0.114, 1.089]; p = 0.016) and shaded outdoor containers (sunlight<20% of the day) (β = 0.538; 95% CI = [0.124, 0.952]; p = 0.011). No difference was detected between shaded outdoor containers and indoor containers (β = 0.063; 95% CI = [−0.413, 0.540]; p = 0.795). Oviposition decreased when containers were covered with lids (β = −0.706; 95% CI = [−1.430, 0.017]; p = 0.056).

**Table 2:** Parameter coefficients for logistic regression model predicting probability of oviposition (n = 591 containers).

Parameter	Regression coefficient	Standard error	z value	Pr>z
Intercept	−0.379	0.247	−1.533	0.125
Larvae (1–10, retained)	1.285^a^	0.362	3.546	**<0.001**
Larvae (1–10, removed)	1.499^a^	0.498	3.011	**0.003**
Larvae (11–50, retained)	1.395^a^	0.415	3.360	**<0.001**
Larvae (11–50, removed)	1.449^a^	0.408	3.550	**<0.001**
Larvae (51–100, retained)	1.937^a^	0.453	4.276	**<0.001**
Larvae (51–100, removed)	2.099^a^	0.616	3.407	**<0.001**
Larvae (>100, retained)	2.332^a^	0.470	4.964	**<0.001**
Larvae (>100, removed)	1.581^a^	0.557	2.840	**0.005**
Location (inside)	−0.601^b^	0.249	−2.416	**0.016**
Location (outside, shade)	−0.538^b^	0.211	−2.550	**0.011**
Lid (present)	−0.706	0.369	−1.914	**0.056**
Collection period 2	−0.788^c^	0.275	−2.863	**0.004**
Collection period 3	−0.209	0.280	−0.749	0.454
Collection period 4	−1.027^c^	0.309	−3.327	**<0.001**

Model was fit using the log-likelihood test to eliminate non-significant predictor variables one at a time (p>0.10). Larvae refers to *Ae. aegypti*. Parameter estimates followed by the same letter are not statistically different from one another as indicated by Tukey's multiple comparisons. Significant p-values are indicated in bold.

**Figure 2:**
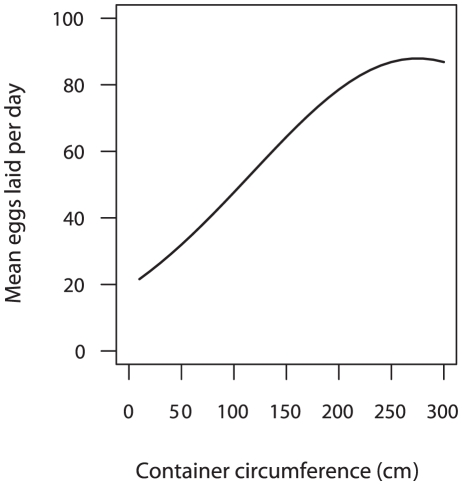
Relationship between eggs laid per day and container circumference. Based on the regression equation y = exp [2.964+(0.011*X)+(−0.00002*X^2^)]. Graph begins at X = 10 cm, the smallest container circumference observed.

Among containers receiving eggs, the number of eggs laid was affected by larval abundance, whether larvae were removed prior to oviposition, pupae, fill method, circumference, and (circumference)^2^ (Table 3). Females laid more eggs when over 50 conspecific larvae were present in containers (β = 0.759; 95% CI = [0.483, 1.035]; p<0.001). Among sites from which larvae were removed prior to egg collection, however, a significant increase in egg abundance was observed only when more than 100 conspecific larvae had been present (β = 0.838; 95% CI = [0.126, 1.549]; p = 0.021). More eggs were laid in containers that held *Ae. aegypti* pupae, regardless of whether they had been removed (β = 0.448; 95% CI = [0.141, 0.754]; p = 0.004). Among the three fill methods, unmanaged containers received a larger number of eggs than rain and manually filled containers (β = 0.387; 95% CI = [0.092, 0.681]; p = 0.010); there was no difference between rain and manual filling (β = 0.073; 95% CI = [−0.241, 0.387]; p = 0.647). Container circumference had a positive effect on egg abundance (β = 0.011; 95% CI = [0.005, 0.017]; p<0.001), whereas the impact of (circumference)^2^ was negative (β = −0.00002; 95% CI = [−0.00003, −0.000004]; p = 0.013). When the regression equation was plotted, egg abundance increased with container size initially, but eventually leveled off as containers approached 270 cm in circumference (Figure 2). No significant interactions were identified between predictor variables in either regression model.

**Table 3:** Parameter coefficients for negative binomial regression model predicting daily number of *Ae. aegypti* eggs laid (n = 306 containers).

Parameter	Regression coefficient	Standard error	z value	Pr>z
Intercept	2.964	0.311	9.543	**<0.001**
Circumference	0.011	0.003	3.633	**<0.001**
(Circumference)^2^	−0.00002	0.000008	−2.478	**0.013**
Larvae (1–10, retained)	0.036	0.220	0.163	0.870
Larvae (1–10, removed)	0.037	0.331	0.113	0.910
Larvae (11–50, retained)	−0.083	0.252	−0.327	0.743
Larvae (11–50, removed)	0.107	0.293	0.365	0.715
Larvae (51–100, retained)	0.846^a^	0.237	3.566	**<0.001**
Larvae (51–100, removed)	0.474	0.353	1.345	0.179
Larvae (>100, retained)	0.784^a^	0.227	3.459	**<0.001**
Larvae (>100, removed)	0.838^a^	0.363	2.308	**0.021**
Pupae (present)	0.448	0.156	2.864	**0.004**
Fill method (manual)	0.073	0.160	0.458	0.647
Fill method (unmanaged)	0.387	0.150	2.578	**0.010**
Collection period 2	−0.470^b^	0.219	−2.144	**0.032**
Collection period 3	−0.531^b^	0.169	−3.138	**0.002**
Collection period 4	−1.293	0.225	−5.754	**<0.001**

Model was fit using the log-likelihood test to eliminate non-significant predictor variables one at a time (p>0.10). Larvae and pupae refer to *Ae. aegypti*. Parameter estimates followed by the same letter are not statistically different from one another as indicated by Tukey's multiple comparisons. Significant p-values are indicated in bold.

### Starvation bioassays

**Figure 3:**
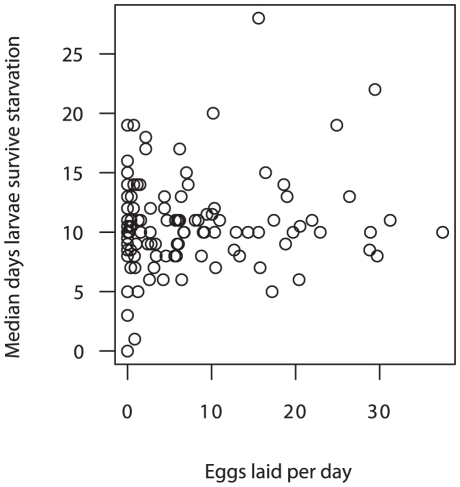
Median resistance to starvation vs. average number of eggs laid. Each circle represents an individual container (n = 113 containers). Median resistance to starvation is the median number of days that larvae from a container survive without food. Number of eggs laid in that container was averaged over the three day survey period and divided by container circumference (mm).

Third instar larvae were collected for starvation bioassays from 113 containers. For the majority of containers, median larval RS was between 5 to 15 days (range 0 to 28 days) (Figure 3). There were no significant correlations between median RS and the mean density of eggs laid per day (all other larvae retained, n = 59 containers, Spearman's ρ = 0.15; all other larvae removed, n = 54 containers, Spearman's ρ = 0.0008). No correlations were evident when the data were also stratified by larval abundance or container capacity (data not shown).

### Experimental study

Ambient temperature and relative humidity were measured for the first four weeks and were consistent among the three study locations (field laboratory, house 1, and house 2) (Table 4). Water temperature (Table 4) was recorded for eight weeks and found to be similar for the field laboratory and house 1. Due to logger malfunction, water temperature was not recorded at house 2. Because Iquitos climate was relatively consistent during June to August 2009 (Table S1), we expect the data recorded at each location to be indicative of the entire study period.

**Table 4:** Air temperature, relative humidity, and water temperature at three experimental study locations.

	Air temperature °C (± SD)				Water temperature °C (± SD)		
Location	Min	Mean	Max	Mean RH % (± SD)	Min	Mean	Max
Field house	24.4±0.7	26.3±0.8	29.2±1.3	82.7±2.5	23.8±0.7	25.4±0.7	27.6±1.2
House 1	24.2±0.8	26.7±1.0	30.9±2.5	82.3±3.0	23.7±0.7	25.3±0.8	27.3±1.2
House 2	23.5±0.7	26.2±0.9	30.6±1.6	84.8±2.6	*	*	*

*Water temperature data are missing from House 2 due to logger malfunction.

**Figure 4:**
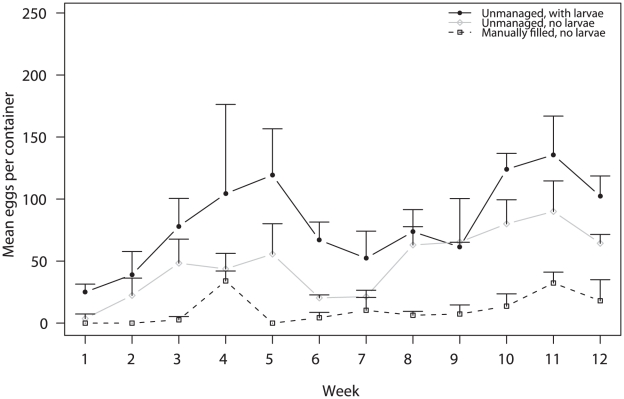
Mean number of eggs (± SE) laid per week by experimental container treatment.

Conspecific larvae were present in treatment A containers and absent from treatment B and C containers throughout the experiment. The number of *Ae. aegypti* eggs laid in each container per week was influenced by container treatment (ANOVA F = 77.70; df = 2, 4; p<0.001) and week (ANOVA F = 6.47; df = 11, 88; p<0.001), but not by house (ANOVA F = 4.45; df = 2, 4; p = 0.096). Females laid the most eggs in unmanaged containers with larvae (A) and the fewest in containers with clean water and no larvae (C) (Figure 4). The number of eggs laid fluctuated over time in all container treatments, peaking in weeks 4 and 5, and again in week 11.

**Figure 5:**
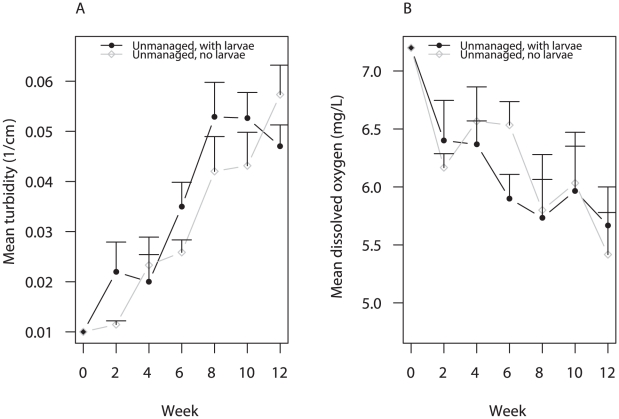
Mean turbidity (± SE) and mean dissolved oxygen (± SE) in unmanaged containers over 12 weeks. Measurements were averaged across all three houses at each time point. Water turbidity (a) was assessed using a 100 cm-long turbidity tube with a Secchi disk affixed to the end. Turbidity was measured as the inverse water depth (1/cm) at which the black and white portions of the Secchi disk were no longer distinguishable. Dissolved oxygen content (b) was measured in mg/L using an Ecological Test Kit (Rickly Hydrological Company, Columbus, OH).

Water in unmanaged containers (A and B) increased in turbidity (ANOVA F = 41.55; df = 6, 30; p<0.001) (Figure 5a) and decreased in dissolved oxygen content over time (ANOVA F = 10.19; df = 6, 30; p<0.001) (Figure 5b), signs of rising levels of organic detritus and microbial growth. Water turbidity and dissolved oxygen content were not influenced, however, by the presence of larvae (treatment A vs. B) (turbidity: ANOVA F = 3.16; df = 1, 2; p = 0.217; oxygen: ANOVA F = 0.19; df = 1, 2; p = 0.704) or location (turbidity: ANOVA F = 5.12; df = 2, 2; p = 0.163; oxygen: ANOVA F = 4.65; df = 2, 2; p = 0.177). Although water assays did not quantify large solid detritus such as leaves, unmanaged containers in each location received similar amounts of detritus due to their proximity to one another. Taken together, our experimental results indicate that food levels were similar among treatment A and B containers, and that differences in oviposition among the two were attributable to the presence of larvae.

## Discussion

In nature, *Ae. aegypti* egg distribution among containers was influenced by a combination of factors, including the presence of conspecific larvae and pupae, container fill method, sun exposure, container size, and the presence of a lid. Although the negative effect of container lid was likely due to presence of a physical barrier [54], consistent patterns with respect to the remaining variables suggest that gravid *Ae. aegypti* females actively choose among potential oviposition sites. Female *Ae. aegypti* responded most strongly to the presence of conspecific immatures, both in terms of the probability of oviposition and the number of eggs laid. This correlation was not due to more frequent presence of adult females in houses with colonized containers. In our study, the presence of colonized containers was not associated with the capture of adult females during entomological surveys. Furthermore, Getis et al. observed a cohort effect among *Ae. aegypti* in Iquitos; infested containers typically held a single cohort of *Ae. aegypti* developing in synchrony, rather than multiple overlapping cohorts [39]. Thus, successive life stages were spatially correlated, but there was no correlation between larval and adult abundance at the household level. After adjusting for conspecific immatures, we did not observe an effect of *Culex* larvae or pupae on *Ae. aegypti* oviposition in our multivariate models.

For *Ae. aegypti*, attraction of gravid females to containers with immature conspecifics may seem at first counter-productive. Field populations are thought to be limited foremost by density-dependent competition for food during the early larval stages [9], [14], [55]. In addition, studies have documented that high larval densities negatively impact several components of mosquito fitness, including larval survivorship [56]–[58], development rate [55], [59], adult lifespan [60], adult size [59], [61], and female fecundity [62], [63]. From this standpoint, it would seem advantageous for ovipositing females to avoid conspecifics as competitors to their own progeny. Interestingly, conspecific attraction has been observed across numerous animal taxa (e.g., reviewed in [64]–[66]), such as birds, mammals, reptiles, fish, and insects, including other mosquitoes [22], [67], [68]. The drawbacks of increased competition may be counter-balanced by the benefit of using conspecifics as a reliable cue of habitat quality [24], [69]. Conspecific attraction has been described as a means for females to exploit information collected by others. Rather than gathering information on a multitude of environmental factors potentially affecting offspring growth, a process constrained by energy, time, and/or sensory capabilities, females may be able to quickly assess habitat suitability by observing the reproductive success of previous females [70]. In the case of *Ae. aegypti*, we speculate that conspecific larvae and pupae may serve as signals that a site experiences infrequent water turn-over and desiccation, and contains adequate food, two conditions necessary for successful larval development.

Due to an inherent trade-off between gaining information on habitat suitability and increasing competition for offspring, we expected conspecific attraction to be tempered by aversion to containers with high larval densities. Laboratory assays have demonstrated a dose-specific oviposition response that increased with conspecific densities up to ∼1 larva/mL and decreased thereafter [28], [71]. In our study, conspecific larvae were always attractive, perhaps because larval densities in Iquitos were far lower (average = 0.03 larvae/mL, SE = 0.006) than the densities found to repel females in laboratory experiments. Only 1.2% of *Ae. aegypti*-colonized containers had densities greater than 1 larva/mL. We suspect that few containers in Iquitos ever reach repellent densities.

We observed that free-ranging *Ae. aegypti* laid more eggs in sites that had recently held conspecifics compared to those that had not, suggesting that conspecific attraction is mediated by chemical cues. The preference for conspecific-conditioned water has been noted in the laboratory [72] and attributed to semiochemicals produced by larval-associated bacteria [71]. Semiochemicals may act as attractants to help females locate cryptic sites, and/or as stimulants to promote egg-laying [30]. Some laboratory studies have revealed preference of ovipositing *Ae. aegypti* for sites containing conspecific eggs [27], [31], leading to the discovery of oviposition-inducing egg semiochemicals [29]. Because our survey required daily collection of eggs, we were unable to investigate in the field the effect of conspecific eggs on *Ae. aegypti* oviposition site selection. Interestingly, when investigators separated the components of these semiochemicals, some components elicited attractive/stimulating responses, whereas others produced repellent/deterrent responses. Depending on their concentration, attractive chemicals can also become repellent [29], [30]. If applied properly, chemical mediators of oviposition behavior have potential to be useful for *Ae. aegypti* control.

Container characteristics such as fill method, sun exposure, and size played a secondary role in oviposition choice. During our observational and experimental studies, more eggs were laid in unmanaged containers and few eggs were laid in manually filled containers. Because unmanaged containers collect the most organic detritus and manually filled containers are kept cleanest, this pattern is consistent with females selecting oviposition sites based on the availability of larval food. If females act primarily to maximize food for their offspring, we would expect the number of eggs laid per container to increase proportionate to food availability. From our starvation assays, however, we were unable to demonstrate any correlation between the median larval survival time, an indirect measure of food availability, and the number of eggs laid per container. Although this result implies that female *Ae. aegypti* did not oviposit to maximize food for their progeny, several limitations of our study could have affected our ability to test this relationship. First, starvation bioassays were conducted on larvae already present in containers at the start of surveys, and thus provided information on container food content over the past few days or weeks, rather than at the moment of oviposition. Because our study design necessitated collecting eggs to quantify oviposition, measuring starvation times of pre-existing third instars was the best alternative. Second, the third instar larvae we collected likely hatched at different time points and results from their starvation bioassays could be confounded by differences in age and time they had to feed.

We also observed more *Ae. aegypti* eggs deposited in containers exposed to sunlight (≥20% of the day). Larval development is highly temperature-dependent [73], [74]. A recent biophysical model of *Ae. aegypti* development in Australia predicted that, when containers are not prone to desiccation, sun-exposed containers reach warmer temperatures and support more generations of *Ae. aegypti* than shaded containers [75]. Females may have a selective advantage if they are able to detect containers with warmer water where their offspring develop faster. This, however, appears to contradict data from Puerto Rico by Barrera et al. [76], who found that immature *Ae. aegypti* were more abundant in shaded containers with low water temperature (≤29°C), indicating that females oviposited more frequently in containers shielded from full sunlight. Due to environmental differences between Iquitos and Puerto Rico, our criteria for shaded vs. exposed may have varied from those used by Barrera et al. [76]. Outdoor containers in Puerto Rico commonly receive sun exposure >50% of the day (ACM, unpublished data), in contrast to Iquitos, where abundant tree coverage limits sun exposure to only 10–40% of the day for most outdoor containers. We cannot directly compare our data to that of Barrera et al. [76] because metrics were not provided for container categories of “full sun,” “partial sun,” or “shaded.” We were not able to measure water temperature in each surveyed container. Maximum daily water temperatures from our experimental containers were typically 27–28°C, suggesting that water temperatures are lower in Iquitos compared to Puerto Rico.

Attraction to large oviposition sites has been demonstrated in *Ae. aegypti* [36] as well as other mosquito species [67], possibly because large sites collect more food or are resistant to desiccation. We found that the number of *Ae. aegypti* eggs laid increased with container circumference up to a threshold around 270 cm, after which oviposition leveled off, indicating that perhaps the relative advantage of large container size diminishes as containers become bigger. Due to the low occurrence in Iquitos of containers exceeding 270 cm in circumference (n = 26 of 591 containers, 4% of surveyed containers), we could not assess the relationship further between increasing container size and oviposition.

A major limitation of our study design was the inability to examine effects of container material and/or texture on oviposition. Container texture affects *Ae. aegypti* oviposition, with females preferring to lay their eggs on rough surfaces [34], [37]. Because we lined containers with strips of paper towel to transport eggs back to the field laboratory, we artificially made container surfaces homogeneous. In a previous Iquitos field study, we showed that females laid more eggs in cement containers compared to plastic or metal containers when all were unlined and similar in size [45]. Additional experimental studies should be conducted to investigate the importance of container material to oviposition site choice when conspecific presence and abundance, fill method, sun exposure, and container size are varied.

*Ae. aegypti* oviposition site choice appears to be flexible, potentially reflecting a mix of site selection strategies across the population. A small portion of females may act as “founders” (e.g., [77]), choosing non-colonized sites based on environmental indicators of quality, whereas the majority of females respond predominantly to conspecific cues. Alternatively, each female may partition her egg batch so that most eggs are laid in colonized containers, when colonized containers are available, and a smaller fraction elsewhere. It should be noted that these scenarios are not mutually exclusive; for any female, the decision to reject or accept a particular site may change with time. For example, results from studies on herbivorous insects demonstrated that ovipositing females typically become more accepting of low-ranking sites as search time progresses (reviewed in [78]). Recent theoretical work on animal decision rules suggests that when individuals are limited by time, number of options, and accuracy with which they can assess site quality, decisions should be based on the best-of-*n* rule [79]. If female *Ae. aegypti* use this rule, they are likely to assess a fixed number of sites (*n*) and choose the perceived best among them, rather than searching longer for a site that meets specific criteria. Such a rule could explain the oviposition patterns we observed in Iquitos; colonized containers tend to be utilized when found, but other site characteristics (size, sunlight, and organic detritus) are used to judge site quality if the *n* sites do not include a colonized container. This remains to be confirmed in the field. Decision rules used by *Ae. aegypti* to select oviposition sites merit further investigation.

Female choice of oviposition site may have greater impact on *Ae. aegypti* population dynamics than previously thought. We propose that, due to strong conspecific attraction, oviposition site selection could lead to dense aggregations of larvae and actually contribute to density-dependent regulation. This phenomenon may explain why larvae in the field frequently develop under food-limiting conditions [9], [38], [80]. It is likely that while some colonized sites become crowded, other suitable larval development sites remain empty. A companion study in Iquitos indicated comparable survival and development rates when larvae were reared in water collected from colonized vs. non-colonized containers in the field, suggesting no difference in food content (STS, unpublished data). These results imply that availability of larval food is not the primary determinant of oviposition choices and agree well with our larval starvation data presented herein. A similar study in Trinidad, West Indies, revealed no difference in nutrient levels between water-storage drums colonized or not by *Ae. aegypti* [81].

Our results have direct implications for strategies to control *Ae. aegypti*. Targeting containers that produce the most *Ae. aegypti* adults for removal or larvicide treatment will reduce mosquito populations in the short term. Sustained population suppression, however, will be difficult to achieve by these means. Elimination of highly productive containers (or the immature *Ae. aegypti* within) will likely shift new eggs to alternative suitable containers. If immature conspecifics are no longer available as a strong oviposition cue, females that would have concentrated their eggs in those highly productive sites may instead oviposit among suitable, previously unoccupied containers based on food availability and/or sun exposure. Strategies that kill mosquitoes late in their development (i.e., insect growth regulators (IGRs) that kill pupae [82], [83] rather than larvae) will enhance vector control by creating “egg sinks,” treated containers that exploit conspecific attraction of ovipositing females, but reduce emergence of adult mosquitoes via density-dependent larval competition and late acting insecticide. For an egg sink strategy, it would be best to employ IGRs that have no repellent effects on ovipositing females, such as pyriproxyfen [84] or methoprene [85]. Pyriproxyfen is of particular interest because adult females are able to transfer the IGR to other oviposition sites [84], [86]. Thus, pyriproxyfen-treated containers could potentially serve as both egg sinks and sources for insecticide dissemination. The success of this approach would depend on oviposition patterns of individual females.

Alternatively, rather than relying on conspecific larvae, control tools could be designed to capitalize on the attractant or stimulant properties of semiochemicals influencing *Ae. aegypti* oviposition responses in the field. Bacteria-derived oviposition attractants could be used to lure females to lethal ovitraps or stimulants could be used to increase their exposure to insecticide-impregnated substrates [30]. The fact that wild *Ae. aegypti* are quite selective when choosing oviposition sites may be the basis for development of new strategies and products for control of dengue virus vectors.

## Supporting Information

Table S1 Mean air temperature, relative humidity, and rainfall in Iquitos by month during 2007 through 2009.
(DOC)

Checklist S1 STROBE checklist.
(DOC)
