# Supplementary material for: Oviposition Site Selection by the Dengue Vector Aedes aegypti and Its Implications for Dengue Control
Source: PLoS Negl Trop Dis. 2011 Apr 12;5(4):e1015. doi: 10.1371/journal.pntd.0001015 (PMC3075222; doi:10.1371/journal.pntd.0001015)
Supplement: Table S1 — Mean air temperature, relative humidity, and rainfall in Iquitos by month during 2007 through 2009. (DOC) [file pntd.0001015.s001.doc]

**Supplemental Information**

**Table S1. Mean air temperature, relative humidity, and rainfall in Iquitos by month during 2007 through 2009.**

| Year | Month | Avg. min. daily temp. ° C ± SD | Avg. max. daily temp. ° C ± SD | Mean temp. ° C ± SD | Mean RH % ± SD | Avg. daily rainfall mm ± SD |
| --- | --- | --- | --- | --- | --- | --- |
| 2007 | January | 22.9 ± 0.5 | 32.2 ± 1.4 | 26.1 ± 0.9 | 83.6 ± 3.5 | 19.8 ± 37.6 |
|  | February | 22.2 ± 0.8 | 34.3 ± 2.0 | 27.2 ± 1.2 | 76.5 ± 6.0 | 4.6 ± 12.4 |
|  | March | 22.6 ± 0.7 | 31.9 ± 1.6 | 25.8 ± 0.8 | 83.2 ± 3.3 | 20.9 ± 36.1 |
|  | April | 22.7 ± 0.8 | 32.1 ± 1.5 | 25.8 ± 1.0 | 83.5 ± 4.2 | 21.8 ± 33.8 |
|  | May | 22.0 ± 1.9 | 31.4 ± 2.2 | 25.1 ± 1.9 | 83.1 ± 3.2 | 15.6 ± 32.2 |
|  | June | 21.6 ± 0.8 | 30.8 ± 1.4 | 25.0 ± 0.7 | 83.6 ± 3.1 | 5.0 ± 10.6 |
|  | July | 20.8 ± 1.9 | 31.3 ± 1.6 | 25.0 ± 1.1 | 80.6 ± 4.4 | 4.9 ± 10.1 |
|  | August | 21.2 ± 0.9 | 32.1 ± 1.3 | 25.5 ± 0.8 | 80.2 ± 4.1 | 7.2 ± 14.9 |
|  | September | 21.7 ± 0.6 | 32.7 ± 1.5 | 25.8 ± 1.0 | 80.5 ± 4.1 | 12.8 ± 26.1 |
|  | October | 22.3 ± 0.8 | 32.5 ± 1.5 | 25.8 ± 1.2 | 81.9 ± 5.2 | 12.9 ± 28.6 |
|  | November | 22.8 ± 0.6 | 32.2 ± 1.6 | 25.9 ± 1.1 | 82.9 ± 4.7 | 18.0 ± 32.6 |
|  | December | 22.9 ± 0.8 | 31.8 ± 1.3 | 26.2 ± 0.9 | 82.1 ± 3.9 | 19.6 ± 48.7 |
| 2008 | January | 22.8 ± 0.6 | 31.2 ± 1.7 | 25.7 ± 1.0 | 82.7 ± 3.8 | 17.8 ± 32.2 |
|  | February | 22.4 ± 0.8 | 31.2 ± 1.8 | 25.7 ± 0.8 | 81.2 ± 5.0 | 11.4 ± 21.8 |
|  | March | 22.7 ± 0.7 | 31.5 ± 1.3 | 25.6 ± 0.9 | 82.2 ± 3.5 | 16.7 ± 30.7 |
|  | April | 22.6 ± 0.8 | 31.8 ± 1.2 | 25.9 ± 0.7 | 82.2 ± 3.1 | 9.0 ± 14.7 |
|  | May | 22.3 ± 1.2 | 30.4 ± 1.6 | 25.3 ± 0.9 | 84.4 ± 3.7 | 8.4 ± 16.5 |
|  | June | 21.4 ± 1.3 | 29.2 ± 2.1 | 24.8 ± 1.1 | 85.5 ± 2.6 | 6.6 ± 13.0 |
|  | July | 21.6 ± 0.7 | 30.9 ± 1.6 | 25.5 ± 0.6 | 84.0 ± 2.9 | 5.1 ± 8.8 |
|  | August | 21.4 ± 1.2 | 34.2 ± 1.5 | 26.3 ± 0.9 | 81.5 ± 4.0 | 3.2 ± 6.6 |
|  | September | 21.6 ± 0.8 | 33.1 ± 2.8 | 26.0 ± 1.6 | 80.4 ± 5.7 | 5.6 ± 11.9 |
|  | October | 22.3 ± 0.9 | 33.5 ± 2.2 | 26.4 ± 1.3 | 81.0 ± 6.0 | 7.1 ± 14.5 |
|  | November | 21.9 ± 0.7 | 32.8 ± 1.8 | 26.3 ± 1.2 | 82.0 ± 4.7 | 7.8 ± 13.5 |
|  | December | 22.3 ± 0.8 | 33.6 ± 1.4 | 26.5 ± 1.1 | 81.1 ± 4.6 | 3.0 ± 6.9 |
| 2009 | January | 22.0 ± 0.6 | 31.9 ± 1.8 | 25.5 ± 0.9 | 86.0 ± 3.9 | 9.5 ± 15.1 |
|  | February | 21.6 ± 0.7 | 33.0 ± 1.5 | 25.9 ± 0.9 | 84.0 ± 4.0 | 9.9 ± 14.4 |
|  | March | 22.5 ± 0.7 | 31.8 ± 2.5 | 25.8 ± 1.0 | 84.5 ± 4.3 | 8.6 ± 16.4 |
|  | April | 22.3 ± 0.7 | 31.6 ± 1.8 | 25.7 ± 1.0 | 86.2 ± 4.4 | 13.0 ± 20.1 |
|  | May | 22.4 ± 0.6 | 32.5 ± 1.7 | 26.2 ± 0.9 | 82.8 ± 4.3 | 4.1 ± 9.6 |
|  | June | 21.5 ± 1.1 | 31.6 ± 1.9 | 25.3 ± 1.0 | 85.0 ± 3.1 | 3.5 ± 5.3 |
|  | July | 21.7 ± 0.6 | 32.0 ± 1.7 | 25.4 ± 0.8 | 84.5 ± 3.4 | 3.7 ± 6.2 |
|  | August | 21.9 ± 0.6 | 33.4 ± 2.0 | 26.4 ± 1.2 | 81.4 ± 4.7 | 3.5 ± 5.6 |
|  | September | 22.2 ± 0.9 | 34.6 ± 1.5 | 26.8 ± 1.0 | 79.8 ± 4.1 | 3.7 ± 8.5 |
|  | October | 22.2 ± 0.9 | 33.8 ± 1.3 | 26.6 ± 1.0 | 81.5 ± 3.9 | 6.7 ± 17.5 |
|  | November | 22.9 ± 0.9 | 34.2 ± 1.4 | 27.0 ± 1.2 | 81.6 ± 4.3 | 7.6 ± 12.3 |
|  | December | 22.7 ± 0.7 | 32.5 ± 1.7 | 26.4 ± 1.2 | 83.7 ± 5.1 | 7.7 ± 12.9 |

Data were collected from a National Oceanic and Atmospheric Administration meteorological station located at the Iquitos airport roughly 6 km from the city center (National Climatic Data Center).

**References Cited**

**National Climatic Data Center, United States Department of Commerce.** Online Climate Data Directory. NCDC, 25 Jun. 2010. Web. Accessed 20 Aug. 2010 <http://www.ncdc.noaa.gov/oa/climate/climatedata.html>.
